# Comparative morphology of the postpharyngeal gland in the Philanthinae (Hymenoptera, Crabronidae) and the evolution of an antimicrobial brood protection mechanism

**DOI:** 10.1186/s12862-015-0565-0

**Published:** 2015-12-21

**Authors:** Katharina Weiss, Erhard Strohm, Martin Kaltenpoth, Gudrun Herzner

**Affiliations:** Evolutionary Ecology Group, Institute of Zoology, University of Regensburg, Universitätsstr. 31, 93053 Regensburg, Germany; Insect Symbiosis Research Group, Max Planck Institute for Chemical Ecology, Hans-Knoell-Str. 8, 07745 Jena, Germany; Department for Evolutionary Ecology, Johannes Gutenberg University Mainz, Institute for Zoology, Johann-Joachim-Becher-Weg 13, 55128 Mainz, Germany

**Keywords:** Postpharyngeal gland, 3D reconstruction, Comparative morphology, Prey preservation, Antimicrobial, Antifungal, Crabronidae, Philanthinae, Beewolves

## Abstract

**Background:**

Hymenoptera that mass-provision their offspring have evolved elaborate antimicrobial strategies to ward off fungal infestation of the highly nutritive larval food. Females of the Afro-European *Philanthus triangulum* and the South American *Trachypus elongatus* (Crabronidae, Philanthinae) embalm their prey, paralyzed bees, with a secretion from a complex postpharyngeal gland (PPG). This coating consists of mainly unsaturated hydrocarbons and reduces water accumulation on the prey’s surface, thus rendering it unfavorable for fungal growth. Here we (1) investigated whether a North American *Philanthus* species also employs prey embalming and (2) assessed the occurrence and morphology of a PPG among females of the subfamily Philanthinae in order to elucidate the evolution of prey embalming as an antimicrobial strategy.

**Results:**

We provide clear evidence that females of the North American *Philanthus gibbosus* possess large PPGs and embalm their prey. The comparative analyses of 26 species from six genera of the Philanthinae, using histological methods and 3D-reconstructions, revealed pronounced differences in gland morphology within the subfamily. A formal statistical analysis based on defined characters of the glands confirmed that while all members of the derived tribe Philanthini have large and complex PPGs, species of the two more basal tribes, Cercerini and Aphilanthopsini, possess simple and comparatively small glands. According to an ancestral state reconstruction, the complex PPG most likely evolved in the last common ancestor of the Philanthini, thus representing an autapomorphy of this tribe.

**Conclusion:**

Prey embalming, as described for *P. triangulum* and *T. elongatus*, and now also for *P. gibbosus*, most probably requires a complex PPG. Hence, the morphology and size of the PPG may allow for inferences about the origin and distribution of the prey embalming behavior within the Philanthinae. Based on our results, we suggest that prey embalming has evolved as an antimicrobial strategy in and is restricted to the tribe Philanthini, which seems to face exceptional threats with regard to fungal infestations of their larval provisions.

**Electronic supplementary material:**

The online version of this article (doi:10.1186/s12862-015-0565-0) contains supplementary material, which is available to authorized users.

## Background

Microorganisms pose serious threats to insects both as pathogens [1–3] and food competitors [4–6]. Many solitary wasps rely on paralyzed arthropod prey as food for their developing offspring [7]. To protect these nutrient-rich resources from harmful microorganisms, wasps have evolved elaborate antimicrobial strategies (e.g. [8–14]). Especially in species that mass-provision their brood with paralyzed prey, it is crucial that the stored resources stay consumable throughout the feeding period of the larva [15].

The European beewolf *Philanthus triangulum* (Fabricius) (Hymenoptera, Crabronidae, Philanthinae) shows a remarkable antimicrobial defense mechanism. Females of this digger wasp prey exclusively on honeybee workers, *Apis mellifera*, as provisions for their offspring (see e.g. [16, 17]). The paralyzed bees are stored in subterranean brood cells under warm and humid conditions, which impose a high risk of fungal infestation on the larval provisions (e.g. [10]). As a countermeasure, females extensively lick the surface of their prey prior to oviposition [10], thereby applying large amounts of a lipid secretion to the bee’s cuticle [18, 19], a behavior termed ‘embalming’ in the following. Since this secretion contains predominantly unsaturated hydrocarbons (HCs) [19, 20], embalming not only increases the total amount of HCs but also the proportion of unsaturated HCs on the prey’s surface [18, 19]. The coating of unsaturated HCs changes the physicochemical properties of the bees’ epicuticle, resulting in a reduction of water condensation. The resulting change in microclimate retards fungal growth, thus reducing the decomposition of the larval resources and increasing larval survival [10, 18, 21].

The source of the embalming secretion is the postpharyngeal gland (PPG) [19, 20, 22], a cephalic gland which has long been thought to be restricted to ants where it is mainly involved in the generation and maintenance of the colony odor ( e.g. [23–26], for a review of other functions see [27]). In female *P. triangulum*, the PPG consists of two large reservoirs originating dorsally from the pharynx at the proximal end of the hypopharyngeal plate and extending laterally anterior to the brain (hereafter referred to as ‘upper part of the PPG’) [22]. Each reservoir of this upper part of the gland consists of a main root with numerous ‘fingers’ branching off, resulting in an overall glove-like shape of the gland [22]. Additionally, a smaller unpaired sac-like evagination extends ventrally from the pharynx (hereafter referred to as ‘lower part of the PPG’) [22]. The walls of all parts of the PPG are formed by a monolayered epithelium with apical hairs that reach into the lumen of the gland. The content of the gland is most probably not synthesized by the epithelial cells but is rather sequestered from the hemolymph via the enlarged gland surface [22, 28].

Besides *P. triangulum*, a PPG has also been described for two species of the genus *Trachypus* [29] (which group within *Philanthus* according to a recent phylogenetic analysis [30]). The morphology of the PPGs of both *Trachypus elongatus* and *Trachypus boharti* closely resembles the PPG of *P. triangulum* [29]. In both *Trachypus* species, the PPG also contains mostly HCs, and *T. elongatus* has been shown to embalm its prey, stingless bees, with the secretion of its PPG [29].

Both species for which prey embalming has so far been described, *P. triangulum* and *T. elongatus*, belong to the tribe Philanthini within the subfamily Philanthinae [30, 31]. The Philanthinae consist of eight genera separated into three tribes [31]: The Philanthini, comprising (*Philanthus* + *Trachypus*) + *Philanthinus*, represent a sister group to the other two tribes, the Cercerini, comprising (*Cerceris* + *Eucerceris*) + *Pseudoscolia*, and the Aphilanthopsini comprising *Clypeadon* + *Aphilanthops* [31]. All philanthine wasps share basic life-history traits, including hunting and nesting behavior. Females build subterranean nests and mass-provision brood cells with paralyzed insects (Hymenoptera or Coleoptera) as food for the developing larvae (e.g. [32–36]). Thus, all Philanthinae may face similar selection pressures with regard to the protection of their larval provisions and their offspring against detrimental microbes. As a consequence, all of these species likely either employ prey embalming with PPG secretion or some other prey preservation mechanism.

In the present study, we aim to shed light on the evolution of the prey embalming behavior and the associated complex PPG and ask whether these traits are common to all Philanthinae or have arisen in only some lineages. First, in order to broaden our knowledge about the distribution of this antimicrobial mechanism within the Philanthinae, we analyzed whether the North American *Philanthus gibbosus* (Fabricius), shows prey embalming. Second, we investigated 26 species belonging to six genera representing all three tribes of the Philanthinae with regard to the occurrence and morphology of the PPG as well as another head gland that could be involved in prey preservation, the mandibular gland (MG). We provide a comparative morphological analysis based on characters obtained by histological investigations and 3D-reconstructions of the head glands. Our analysis revealed pronounced differences in the morphology of the PPG between the different tribes of the Philanthinae, which may allow for inferences about the origin and distribution of the prey embalming behavior within this subfamily.

## Methods

### Prey embalming in *Philanthus gibbosus*

A detailed description of the methodology of this section is given in Additional file 1. Briefly, *P. gibbosus* females were reared in observation cages as described earlier for *P. triangulum* [37] and supplied with halictid bees (Hymenoptera, Halictidae) as prey. Owing to the limited availability of halictid bees, several different species had to be used. To investigate whether *P. gibbosus* females embalm their prey with HCs from their PPG, paralyzed bees were removed from *P. gibbosus* brood cells (hereafter referred to as ‘provisioned bees’, *N* = 6) and their cuticular HCs were analyzed by gas chromatography/mass spectrometry (GC/MS). For comparison, halictid bees which had no contact to the beewolves (hereafter referred to as ‘control bees’, *N* = 9) and heads of field-caught *P. gibbosus* females (*N* = 4) were analyzed accordingly. As both provisioned and control bees comprised a number of different halictid species, a molecular identification of the species was conducted [see Additional file 1: Table S1]. For the chemical analysis, whole bees as well as *P. gibbosus* heads were extracted, an internal standard was added for quantification and aliquots were analyzed by GC/MS. *N*-alkanes were identified by comparison of their retention times and mass spectra to synthetic references. Linear retention indices (LRIs) for all other substances were calculated in relation to the *n*-alkanes [38], and alkenes were identified by their LRIs and mass spectra as described in Strohm *et al.* [20]. The structure of the unsaturated ketone nonacosen-6-one was tentatively assigned by its mass spectrum as described previously [29]. Absolute amounts of components were calculated by use of the internal standard and compared between provisioned and control bees by a Mann–Whitney *U* test. Relative amounts were calculated by standardizing the total peak area of a sample to 100 %. The proportions of unsaturated compounds were *arcsine*-transformed and compared between provisioned and control bees using a *t* test; in addition, we compared the relative amounts of individual HCs. All tests were performed using the statistics software package PAST (Version 2.08b) [39]. Unless otherwise stated, values given are means ± standard deviation (SD).

### Comparative morphology of head glands

#### Specimens

Females of 26 species and subspecies belonging to six genera covering the three tribes of the crabronid subfamily Philanthinae were included in the morphological analysis (Table 1). The tribe Philanthini was represented by eight *Philanthus* species from Europe and South Africa, nine *Philanthus* species from North America, four *Trachypus* species from South America, and one *Philanthinus* species from Turkey. The Aphilanthopsini were represented by one *Clypeadon* and one *Aphilanthops* species from North America, and the Cercerini by two European *Cerceris* species.

**Table 1:** Philanthine species included in the comparative morphological study

Species	N	Country	Fixative	3D
*Aphilanthops frigidus*	2	USA	AAF	yes
*Clypeadon laticinctus*	3	USA	AAF	yes
*Cerceris arenaria*	2	Germany	Bouin	yes
*Cerceris quinquefasciata*	3	Germany	AAF	yes
*Philanthinus quattuordecimpunctatus*	3	Turkey	EtOH	yes
*Trachypus flavidus*	1	Brazil	EtOH	yes
*Trachypus elongatus*	3	Brazil	AAF	yes
*Trachypus patagonensis*	1	Brazil	AAF	no
*Trachypus boharti*	3	Brazil	AAF	yes
*Philanthus venustus*	1	Turkey	AAF	yes
*Philanthus coronatus*	1	Germany	Bouin	yes
*Philanthus triangulum*	3	Germany	Bouin	no
*Philanthus triangulum diadema*	3	South Africa	AAF	yes
*Philanthus capensis*	1	South Africa	AAF	yes
*Philanthus loefflingi*	3	South Africa	AAF	yes
*Philanthus rugosus*	3	South Africa	AAF	yes
*Philanthus melanderi*	1	South Africa	AAF	yes
*Philanthus bicinctus*	1	USA	Bouin	yes
*Philanthus ventilabris*	2	USA	AAF	yes
*Philanthus crabroniformis*	1	USA	AAF	no
*Philanthus multimaculatus*	1	USA	AAF	yes
*Philanthus barbiger*	2	USA	AAF	yes
*Philanthus gibbosus*	3	USA	Bouin	yes
*Philanthus albopilosus*	2	USA	Bouin	yes
*Philanthus psyche*	2	USA	AAF	yes
*Philanthus pulcher*	1	USA	AAF	no

List of species included in the comparative morphological study of head glands of female Philanthinae, including information on the number of specimens examined (N), collection site of the species (Country), the fixative agent used for the histological preparation (Fixative; AAF = formalin-ethanol-acetic acid; Bouin = alcoholic Bouin; EtOH = 100 % ethanol), and whether a 3D-reconstruction of the head glands was conducted (3D; yes = 3D-reconstruction; no = glands not reconstructed)

#### Histology

Wasps were cold anesthetized and decapitated. The heads were fixed using different fixing agents (Table 1). For large heads, both compound eyes were laterally cut off after fixation using sharp razor blades to facilitate the infiltration of the embedding medium. Heads were then thoroughly rinsed in 70 % or (in case of formalin-ethanol-acetic acid fixation) 80 % ethanol, dehydrated in a graded ethanol series and propylene oxide, and embedded in Epon 812 according the suppliers instructions (Polysciences Europe GmbH, Eppelheim, Germany). Sagittal semithin sections (4 μm) were cut with a microtome (Reichert Ultracut; Leica Microsystems AG, Wetzlar, Germany) equipped with a diamond knife and subsequently stained with toluidine blue [40]. The resulting series of histological sections were used for histological investigation of the head glands by light microscopy (Zeiss Axiophot 2; Carl Zeiss Microscopy GmbH, Jena, Germany) using bright field and differential interference contrast settings and for 3D-reconstructions of the head glands.

### 3D-reconstruction

To visualize the overall morphology of head glands and facilitate their comparison, 3D-reconstructions of the head glands were conducted for 22 of the 26 investigated species. Even though for one *Trachypus* and three *Philanthus* species no complete series of sections were available (Table 1), the histological sections were sufficient to allow for the determination of gland characters. For 3D-reconstruction, continuous series of semithin sections of one individual per species were photographed using a digital microscope camera (Olympus DP20; Olympus, Japan) attached to a light microscope (Zeiss Axiophot 2; Carl Zeiss Microscopy GmbH, Jena, Germany). The digitalized sections were automatically aligned with regard to each other using the software package TrakEM2 [41] for the open source image processing software Fiji [42], and the alignment was subsequently corrected manually where necessary. The relevant structures within the head capsule were then marked as 3D-objects in TrakEM2 by manually outlining them in each picture of a series. Finally, 3D-reconstructions were calculated and visualized using Fiji’s 3D-viewer plug-in [43].

### Statistical analysis of gland morphology

After a comprehensive examination of both semithin histological sections and 3D-reconstructions of the head glands of female Philanthinae, we defined eight morphological characters of the PPG for a comparative statistical analysis. As the PPG might not be the only possible source of the embalming secretion (see Discussion), we also included five morphological characters of the MG, the only other head gland of female Philanthinae with a reservoir of considerable size. The 13 characters of the two glands comprised information on gland structure, as well as their overall shape and location within the head capsule. For each character, the different character states were categorized and numerically coded for statistical analysis. Detailed descriptions of all characters and the coding of their different states are given in Additional file 1. In short, the defined characters were: (1) overall structure of the PPG, (2) shape of the upper part of the PPG, (3) number of lobes of the upper part of the PPG, (4) number of openings of the upper part of the PPG to the pharynx, (5) relative size of the PPG in relation to the head capsule, (6) structure of the inner walls of the PPG, (7) shape of the lower part of the PPG, (8) number of openings of the lower part of the PPG to the pharynx, (9) overall structure of the MG, (10) relative size of the MG in relation to the head capsule, (11) branching of the MG, (12) structure of the inner walls of the MG, (13) association of the MG with gland cells.

To formally assess the pattern of PPG morphology among the species we used characters 1 through 8 of the data matrix [see Additional file 1: Table S2] to conduct a hierarchical cluster analysis for ordination with the statistics software package PAST (Version 2.08b) [39]. In a second analysis, the morphological characters of the MG were included [see Additional file 1: Table S2, characters 9 through 13] in order to consider a possible impact of MG morphology on the clustering of species. Bray-Curtis indices were used as similarity measures and ‘unweighted pair-group averages’ as the clustering algorithm; the number of bootstrap replicates was set to 10,000.

To trace the evolution of PPG overall shape, the character which shows the most striking differences among the Philanthinae and most probably reflects differences in function of the PPG (see Results und Discussion), we conducted an ancestral state reconstruction [44] based on the molecular phylogeny of the Philanthinae [30]. The ancestral state reconstruction was performed using both maximum parsimony (unordered character states) and maximum likelihood (ML) approaches in Mesquite (Version 3.04) [45]. The ML analysis was performed using the Markov k-state 1 parameter model, in which any particular character change is considered equally probable, as the model of evolution. As outgroup we included the only apoid wasp outside the Philanthinae currently known to possess a PPG, the cockroach wasp *Ampulex compressa* (Fabricius) (Hymenoptera, Ampulicidae) [46]. In *A. compressa* the PPG is located behind the brain (as opposed to is location anterior to the brain in the Philanthinae), but it shows the typical structure (monolayered epithelium, hairs), ultrastructure of the epithelial cells, and chemistry (HCs) [46] that can be found in both, the Philanthinae [20, 22, 29] and ants [23–27]. With reference to a recent concept of homology [47], which proposes that the position of homologous characters can be variable among species, we tentatively assume homology between the PPGs of *A. compressa* and the Philanthinae.

## Results

### Prey embalming in *Philanthus gibbosus*

To establish whether *P. gibbosus* females embalm their prey, the chemical profiles of *P. gibbosus* females’ heads, surfaces of provisioned bees taken from *P. gibbosus* brood cells, and those of control bees caught in the field were compared. In head extracts of *P. gibbosus* females, we detected 19 compounds, including 17 alkanes, alkenes, and alkadienes with chain lengths ranging from 23 to 31 carbon atoms, as well as the unsaturated long-chain ketone nonacosen-6-one, and one unidentified compound [see Additional file 1: Table S3]. The extracts were dominated by unsaturated HCs (mean proportion 87 ± 2 %), with the main compounds being pentacosene (24 ± 6 %), heptacosene (31 ± 3 %), nonacosene (44 ± 3 %), and hentriacontene (3 ± 0.8 %), as well as the alkane pentacosane (30 ± 3 %). Among the minor compounds was the alkene octacosene. The extracts of control bees contained 56 substances, of which 32 were identified as alkanes, alkenes, and alkadienes; the remaining 24 compounds were not further identified [see Additional file 1: Table S3]. The samples of provisioned bees contained a total of 45 substances, including 27 alkanes, alkenes, and alkadienes, as well as the ketone nonacosen-6-one, and 16 unidentified substances [see Additional file 1: Table S3].

**Fig. 1:**
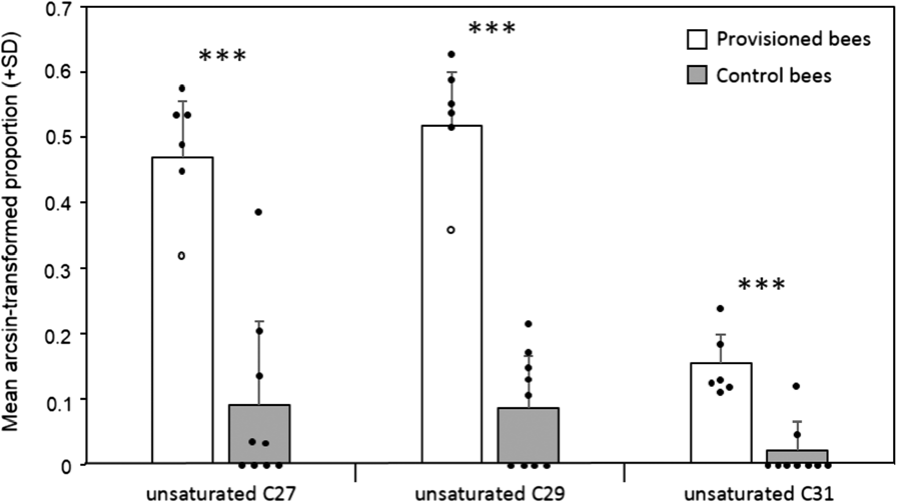
Major hydrocarbons of *P. gibbosus* on provisioned and control bees. Mean *arcsine*-transformed proportion (+ standard deviation, SD) of heptacosene + heptacosadiene (unsaturated C27), nonacosene (unsaturated C29), and hentriacontene + hentriacontadiene (unsaturated C31) in extracts of provisioned bees taken from *P. gibbosus* brood cells (white bars, *N* = 6) and control bees which had no contact to the beewolves (grey bars, *N* = 9); closed circles show individual data points (open circles indicate outliers). Asterisks indicate a significance level of *p ≤* 0.001 according to *t* tests (including outliers)

The two minor compounds octacosene and nonacosen-6-one occurred only in extracts of *P. gibbosus* females and provisioned bees, but not in those of control bees [see Additional file 1: Table S3]. Moreover, the proportions of the major compounds of *P. gibbosus* females (sum of isomers if more than one occurred) were significantly higher on provisioned than on control bees (Fig. 1): heptacosene + heptacosadiene (21 ± 6 % *vs.* 2 ± 5 %; *t* test: t = 5.93, *p <* 0.0001), nonacosene (25 ± 7 % *vs.* 1 ± 1 %; *t* test: t = 9.4, *p <* 0.0001), and hentriacontene + hentriacontadiene (3 ± 2 % *vs.* 0.2 ± 0.4 %; *t* test: t = 5.39, *p =* 0.0001).

**Fig. 2:**
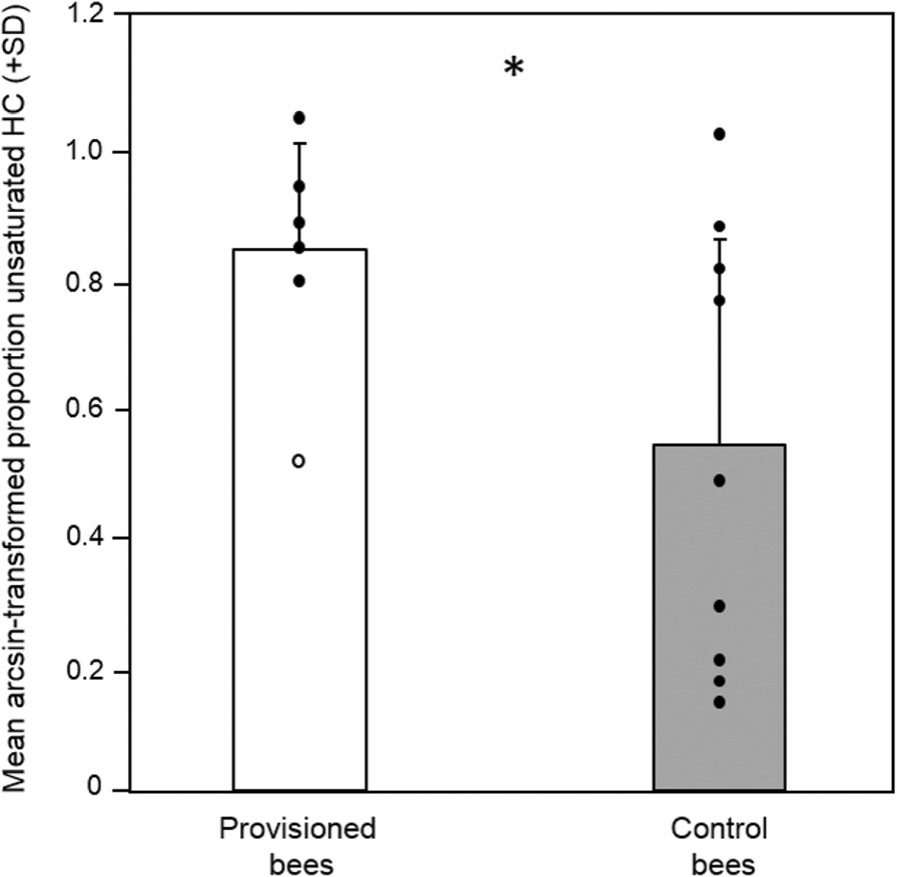
Proportion of unsaturated hydrocarbons on provisioned and control bees. *Arcsine*-transformed proportion (+ standard deviation, SD) of unsaturated hydrocarbons (HCs) in extracts of provisioned bees (*N* = 6) taken from *P. gibbosus* brood cells and control bees (N = 9) which had no contact to the beewolves; closed circles show individual data points (open circle indicates outlier). Asterisk indicates a significance level of *p <* 0.05 according to a *t* test (including outlier)

Most importantly, provisioned bees carried a considerably larger proportion of unsaturated HCs than controls (55 ± 16 % *vs.* 31 ± 26 %; *t* test: t = 2.3, *p =* 0.04; Fig. 2). The total amount of cuticular substances on provisioned bees was, on average, twice as high as on control bees; however, this difference was statistically not significant (8.7 ± 6.5 μg *vs.* 4.1 ± 3.9 μg; Mann–Whitney* U* test, N provisioned bees = 6, N controls = 9, *U* = 12, exact *p =* 0.09).

### Comparative morphology of head glands

**Fig. 3:**
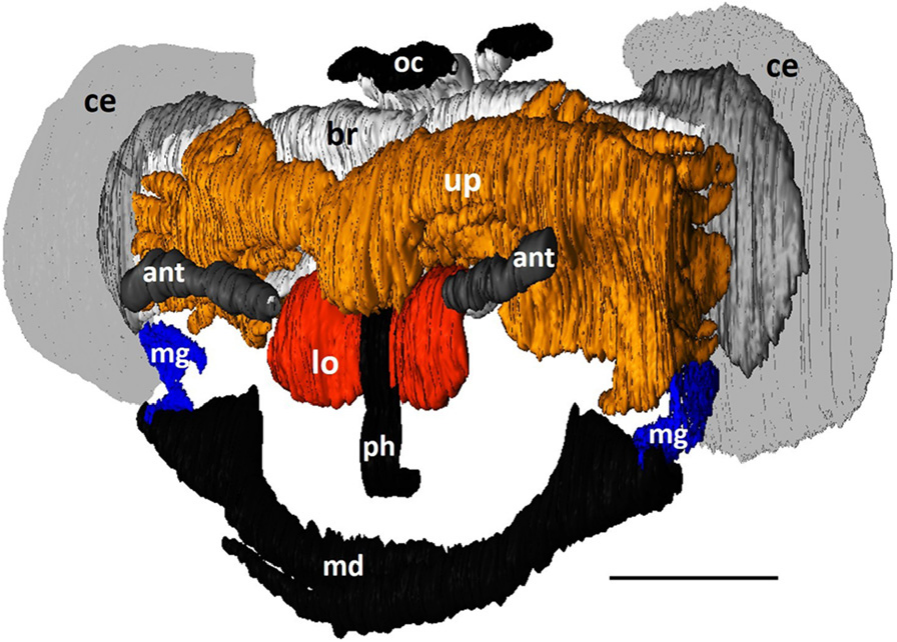
3D-reconstruction of the head structures of a female *Philanthus barbiger*. The upper reservoir of the PPG (orange) is located in front of the brain (light grey) and extends laterally towards the compound eyes (grey), the sac-like lower reservoir of the gland (red) originates ventrally from the pharynx (black). The small MG reservoirs (blue) are located laterally on both sides of the head capsule, opening at the mandibular base. Abbreviations: ant, antenna; br, brain; ce, compound eye; lo, lower part of PPG; md, mandibles; mg, mandibular gland reservoir; oc, ocelli; ph, pharynx; up, upper part of PPG. Scale bar = 0.5 mm

**Fig. 4:**
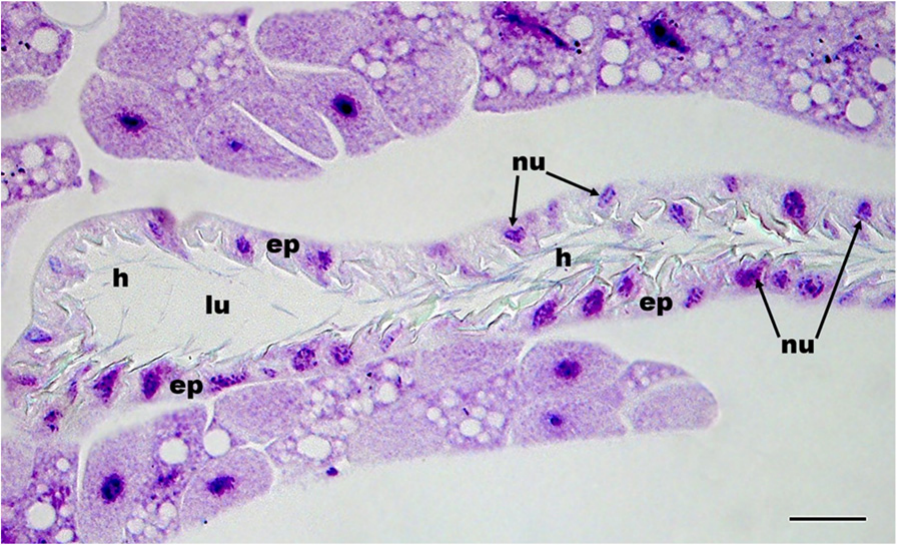
Histological section of the postpharyngeal gland (PPG) of a female *Philanthus rugosus*. The epithelial cells of the upper reservoir of the PPG bear cuticular hairs reaching into the lumen of the gland. Abbreviations: ep, epithelial cells; h, hairs; lu, lumen of the upper PPG reservoir; nu, nuclei of epithelial cells. Scale bar = 25 μm

Females of all 26 investigated philanthine species possessed evaginations of the pharynx, originating posterior to the hypopharyngeal plate and, thus, qualifying as PPGs (Fig. 3, *Philanthus barbiger*). In all species, the wall of the PPG was formed by a monolayered epithelium, whose cells bore hair-like structures on their inner (apical) sides (Fig. 4). The density of these hairs varied between species [see Additional file 1: Table S2, character 6] and was generally found to decline from the proximal part near the pharynx to the distal parts of the lateral branches of the gland. Besides the paired upper part of the PPG, most species also possessed a smaller sac-like lower part of the gland originating ventrally from the pharynx [see Additional file 1: Table S2, character 1].

**Fig. 5:**
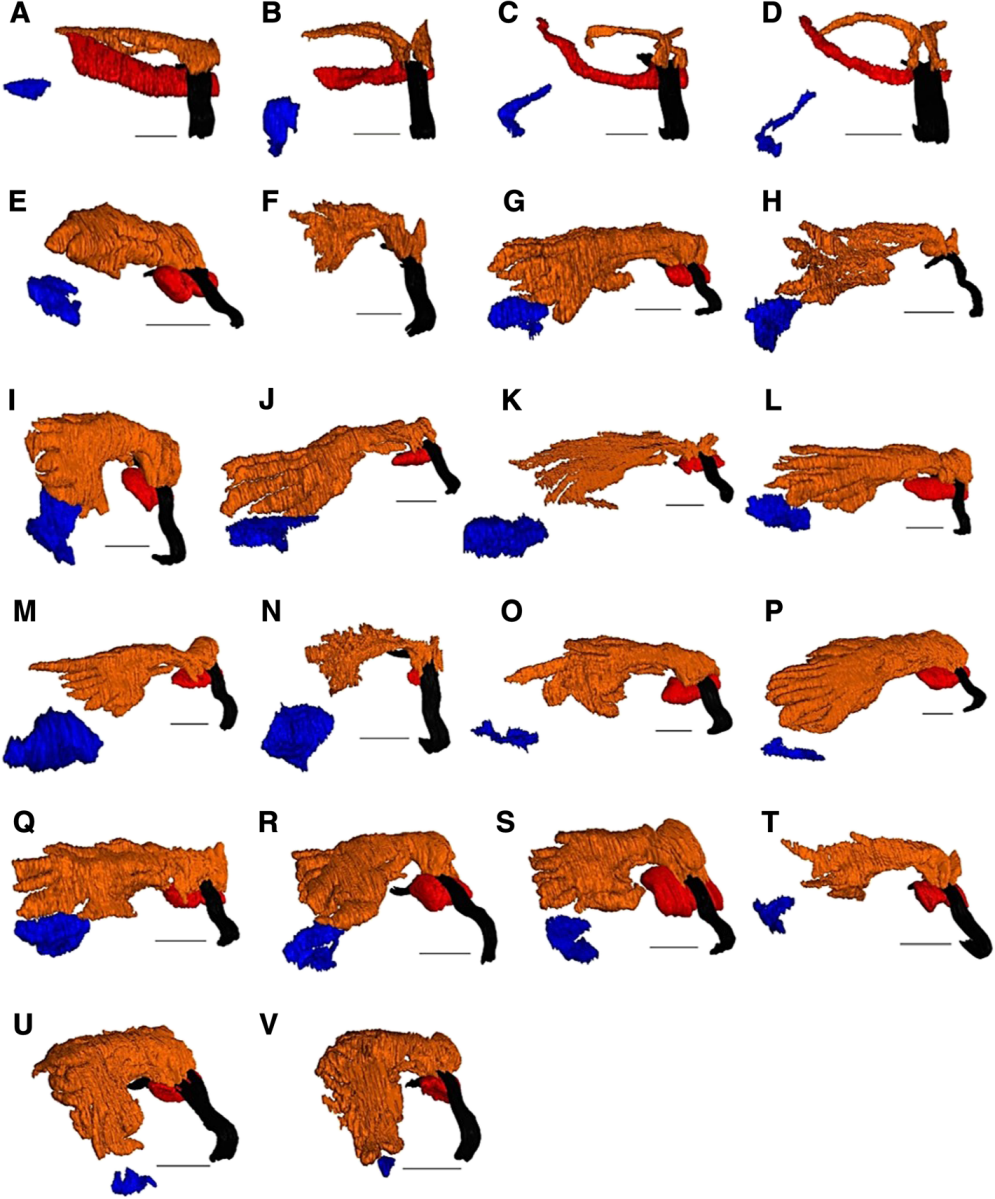
3D-reconstructions of the head glands of female Philanthinae. The postpharyngeal gland (PPG) is shown in orange and red; the mandibular gland (MG) is shown in blue. Note that for each species only the right part of the head capsule is shown. **a** *Aphilanthops frigidus*; **b** *Clypeadon laticinctus*; **c** *Cerceris arenaria*; **d** *Cerceris quinquefasciata*; **e** *Philanthinus quattuordecimpunctatus*; **f** *Trachypus flavidus* (note that for this species the MG could not be reconstructed based on the available serial histological sections); **g** *Trachypus elongatus*; **h** *Trachypus boharti*; **i** *Philanthus venustus*; **j** *Philanthus triangulum diadema*; **k** *Philanthus capensis*; **l** *Philanthus loefflingi*; **m** *Philanthus rugosus*; **n** *Philanthus melanderi*; **o** *Philanthus coronatus*; **p** *Philanthus bicinctus*; **q** *Philanthus ventilabris*; **r** *Philanthus multimaculatus*; **s** *Philanthus barbiger*; **t** *Philanthus gibbosus*; **u** *Philanthus albopilosus*; **v** *Philanthus psyche*. Color code: orange, upper part of the PPG; red, lower part of the PPG; blue, MG; black, pharynx. Scale bars = 0.25 mm

The 3D-reconstructions revealed two distinct ‘types’ of overall PPG morphology: In all Aphilanthopsini (*Aphilanthops frigidus*, *Clypeadon laticinctus*) and Cercerini (*Cerceris arenaria*, *Cerceris quinquefasciata*) each side of both upper and lower part of the PPG consisted of a simple tube-shaped evagination of the pharynx, with the glands of *Aphilanthops* and *Clypeadon* being somewhat stouter than those of *Cerceris* (Fig. 5a-d). Except for *A. frigidus*, where the two sides of the upper part of the PPG shared one opening, each PPG-tube had a separate opening to the pharynx [see Additional file 1: Table S2, characters 4 and 8].

By contrast, in the 17 investigated species and subspecies of the tribe Philanthini (*Philanthus*, *Trachypus*, and *Philanthinus*) the upper part of the PPG was comparatively larger and showed a glove-or comb-like overall structure with multiple ‘fingers’ branching off from a common root (Fig. 5 e-x). The number of these ‘fingers’ varied among species, ranging from seven to more than 15 per side [see Additional file 1: Table S2, character 3]. The left and right part of the upper PPG had either separate openings to the pharynx or shared a common opening [see Additional file 1: Table S2, character 4]. The lateral extension of the upper part of the PPG varied among species, ranging from 55 % to 76 % of the head capsule width [see Additional file 1: Table S2, character 5]. Two of the investigated *Trachypus* species, *T. flavidus* and *T. boharti*, lacked the lower part of the PPG (Fig. 5 f and h). In the other species of the Philanthini, the lower part of the PPG consisted of an unpaired sac-like evagination that was considerably smaller than the upper part of the gland.

**Fig. 6:**
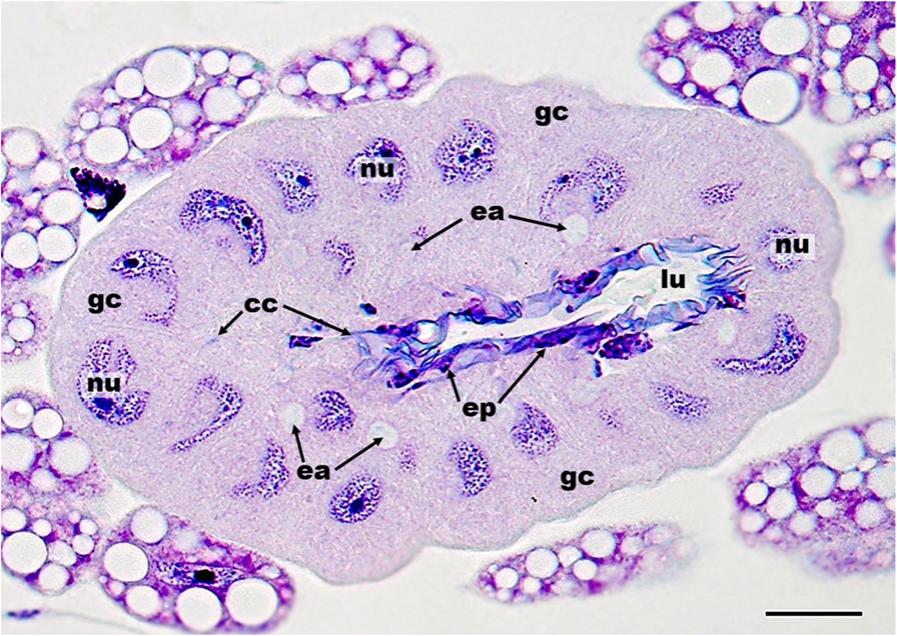
Histological section of the mandibular gland (MG) of a female *Cerceris quinquefasciata*. The class III gland cells associated with the MG can be identified by their end apparatuses and conducting canals. Abbreviations: cc, conducting canals of class III gland cells; ep, epithelial cells of the MG reservoir; gc, class III gland cells; lu, lumen of the MG reservoir; nu, nuclei of class III gland cells. Scale bar = 25 μm

In comparison to the PPG, the differences in the overall morphology of the MG among species were smaller. The MG of all investigated Philanthinae consisted of a pair of lateral and (relative to the PPG) small, unbranched sac-like reservoirs that opened dorsally at the base of the mandibles (Fig. 3) [see Additional file 1: Table S2, characters 9 and 11]. The walls of the MG were formed by a monolayered epithelium, which did not bear any conspicuous surface structures in any species (Fig. 6) [see Additional file 1: Table S2, character 12]. The size of the MG, however, varied somewhat among species (Fig. 5) [see Additional file 1: Table S2, character 10]. In the two investigated *Cerceris* species, the six *Philanthus* species *P. triangulum*, *P. triangulum diadema, P. gibbosus*, *Philanthus melanderi*, *Philanthus coronatus*, and *Philanthus albopilosus*, as well as in *T. boharti* and *T. flavidus* the MG reservoir was associated with class III gland cells (classification according to Noirot and Quennedey [48]; Fig. 6), which were identified by their conspicuous end apparatuses and conducting canals. Other cells surrounding the MG in other species might constitute class I gland cells [48]; however, these cells could not unambiguously be identified as secretory cells with the light-microscopic methods applied.

**Fig. 7:**
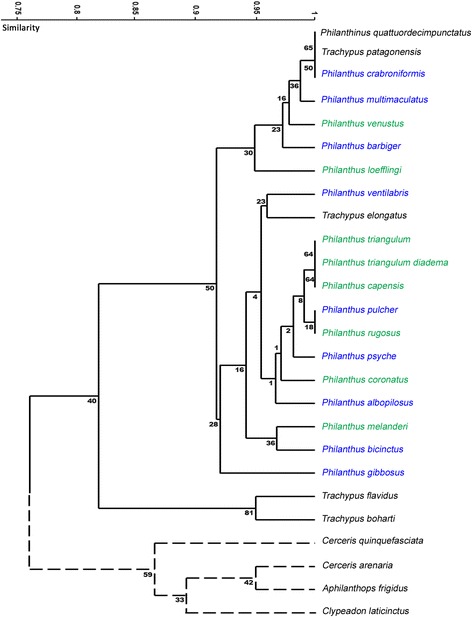
Dendrogram of Philanthinae based on postpharyngeal gland morphology. Dendrogram obtained by a hierarchical cluster analysis based on eight morphological characters of the postpharyngeal gland (PPG) of female Philanthinae. Values at the nodes are bootstrap values. Solid lines indicate members of the tribe Philanthini, dashed lines indicate members of the tribes Cercerini and Aphilanthopsini. Names of European and South African *Philanthus* species are printed in green; names of North American *Philanthus* species are printed in blue. Bray-Curtis was used as similarity index; N bootstrap replicates = 10,000

The 13 morphological characters defined for the PPG and MG are summarized in the data matrix displayed in Table S2 [see Additional file 1]. A hierarchical cluster analysis using the eight characters defined for the PPG (Table S2, characters 1-8) formally confirmed the results obtained by the histological examination and 3D-reconstructions of the PPGs (Fig. 7). The first bifurcation of the dendrogram separated all species of Philanthini, possessing big glove-shaped PPGs, from both Cercerini and Aphilanthopsini, with simple tube-shaped PPGs. Within the Philanthini, the second bifurcation separated *T. flavidus* and *T. boharti*, which do not possess the lower sac-like evagination of the PPG, from all other Philanthini. The congeneric *T. patagonensis* and *T. elongatus*, however, clustered deeply within the other Philanthini. Generally, no obvious grouping of the species within a tribe could be observed based on PPG morphology. Moreover, most nodes were supported by only low bootstrap values. The inclusion of the data on MG morphology altered the location of single species within the Philanthini, but did not change the basic results obtained by the analysis of the PPG morphology alone [see Additional file 1: Figure S1].

**Fig. 8:**
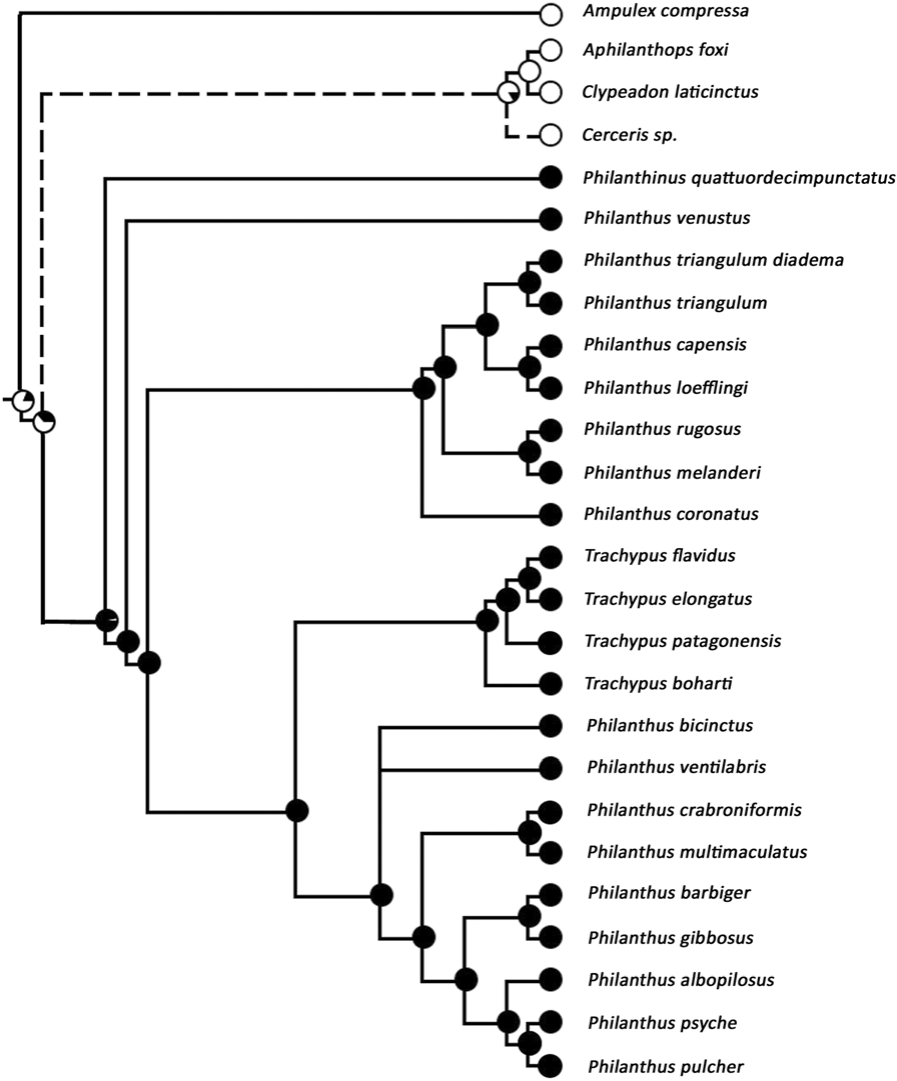
Maximum likelihood (ML) ancestral state reconstruction of the overall shape of the postpharyngeal gland in the Philanthinae. The dendrogram is based on a molecular phylogeny; dashed lines indicate members of the philanthine tribes Cercerini and Aphilanthopsini. ML ancestral state reconstructions, using the Markov k-state 1 parameter model, for each node are visualized using pie charts; color code: white, simple tube-shaped PPGs; black, complex branched PPGs. As both maximum parsimony and ML analyses yielded the same results, only the ML data are shown. Note that the molecular phylogeny comprised *Aphilanthops foxi* and one unidentified *Cerceris* species as representatives of the respective genera

The ancestral state reconstruction showed that the simple tube-shaped PPGs of Cercerini and Aphilanthopsini most likely are the ancestral PPG ‘type’ in the Philanthinae (ML probability: 66 %), while the more complex glove-shaped PPG most likely evolved in the last common ancestor of the tribe Philanthini (ML probability: 96 %) (Fig. 8).

## Discussion

### Prey embalming in *Philanthus gibbosus*

We provide clear evidence that female *P. gibbosus*, as has been described for *P. triangulum* [10, 19] and *T. elongatus* [29], embalm their prey with HCs from their PPG: (1) Head extracts of female *P. gibbosus* contained predominately unsaturated HCs, like the PPGs/heads of *P. triangulum* [20], *T. elongatus*, and *T. boharti* [29], (2) the two components octacosene and nonacosen-6-one occurred only in samples of *P. gibbosus* heads and provisioned bees from their nests, but not on control bees, (3) the proportion of unsaturated HCs was significantly higher on provisioned bees than on control bees, and (4) this increase in the proportion of unsaturated HCs on provisioned bees was mostly due to an increase in the proportion of the major unsaturated HCs of *P. gibbosus* females.

Although the total amount of cuticular HCs was on average twice as high on provisioned bees as on control bees, the difference was not statistically significant. This may be due to the high variance in the total amount of substances within the two groups, which is partly explained by the fact that different halictid species were included in the analysis. The proportion of unsaturated compounds, on the other hand, was significantly higher on provisioned bees than on controls. Similar results (i.e. a significant difference with regard to the proportion of unsaturated HCs but not the total amount of HCs on provisioned *vs.* control bees) have been obtained for *T. elongatus* [29]. In *P. triangulum*, by contrast, the prey embalming results in a significant increase in both the total amount of cuticular HCs and the proportion of unsaturated components [18, 19]. As has been demonstrated in *P. triangulum*, it is the increased proportion of unsaturated HCs, rather than the total amount of cuticular substances, that is crucial for the change in physicochemical properties and the resulting antimicrobial effect of prey embalming [18]. Thus, we assume that *P. gibbosus* employs a similar mechanism to protect its prey from molding as *P. triangulum* and *T. elongatus*.

According to a recent molecular phylogeny of the Philanthinae [30], several clades can be distinguished within the tribe Philanthini that generally coincide with their geographical distribution: The genus *Philanthinus* constitutes the most basal taxon of the tribe. The North American *Philanthus* and the South American *Trachypus* are sister taxa (rather than *Trachypus* being a separate genus, [30]) that group within the European and African *Philanthus* species. The fact that the three species that have yet been shown to embalm their prey (the Afro-European *P. triangulum,* the North American *P. gibbosus* and the South American *T. elongatus*) represent the three major clades of the Philanthini suggests that prey embalming is widespread at least within this tribe.

### Comparative morphology of head glands

All three species that have been shown to employ prey embalming (*P. triangulum*, *P. gibbosus*, and *T. elongatus*) have rather large and complex PPGs. The PPG of female *P. triangulum* contains a mean amount of 330 μg of secretion (maximum: 1,400 μg) [20], of which approximately 80 to 110 μg are applied to a single prey item [18, 19]. Even though the other two species seem to apply less secretion onto their prey, it stands to reason that a large and complex PPG is a prerequisite for prey embalming. The occurrence, morphology, and size of the PPG may hence allow for inferences about the origin and distribution of the prey embalming behavior within the subfamily Philanthinae.

All 26 philanthine species under study, including members of the hitherto not investigated genera *Philanthinus*, *Cerceris*, *Clypeadon*, and *Aphilanthops*, possess head glands that can be classified as PPGs due to their location and basic morphology. As in *P. triangulum* [22], *T. elongatus*, and *T. boharti* [29], the PPGs of the newly investigated species consist of paired evaginations of the pharynx anterior to the brain, constituting the upper part of the PPG, and an either paired or unpaired lower part (which is missing in some species). In all cases, the gland reservoir is bordered by a monolayered epithelium, the cells of which bear conspicuous hairs that reach into the lumen of the gland.

However, the histological investigation and 3D-reconstructions of the PPGs also revealed distinct differences in the morphology of the PPG between the tribes: The PPGs of all members of the Philanthini (*Philanthus, Trachypus*, and *Philanthinus*) are rather uniform in that they possess a voluminous upper part consisting of multiple ‘fingers’ branching off from a common root and the lower part comprising a considerably smaller unpaired sac-like evagination of the pharynx, thus, closely resembling the PPGs of *P. triangulum* [22], as well as *T. elongatus* and *T. boharti* [29].

The structure of the PPG in females of the Cercerini and Aphilanthopsini differed markedly in that both upper and lower part of the PPG are paired tube-shaped evaginations of the pharynx that do not show any branching. Moreover, the glands are smaller than those found in the Philanthini.

**Fig. 9:**
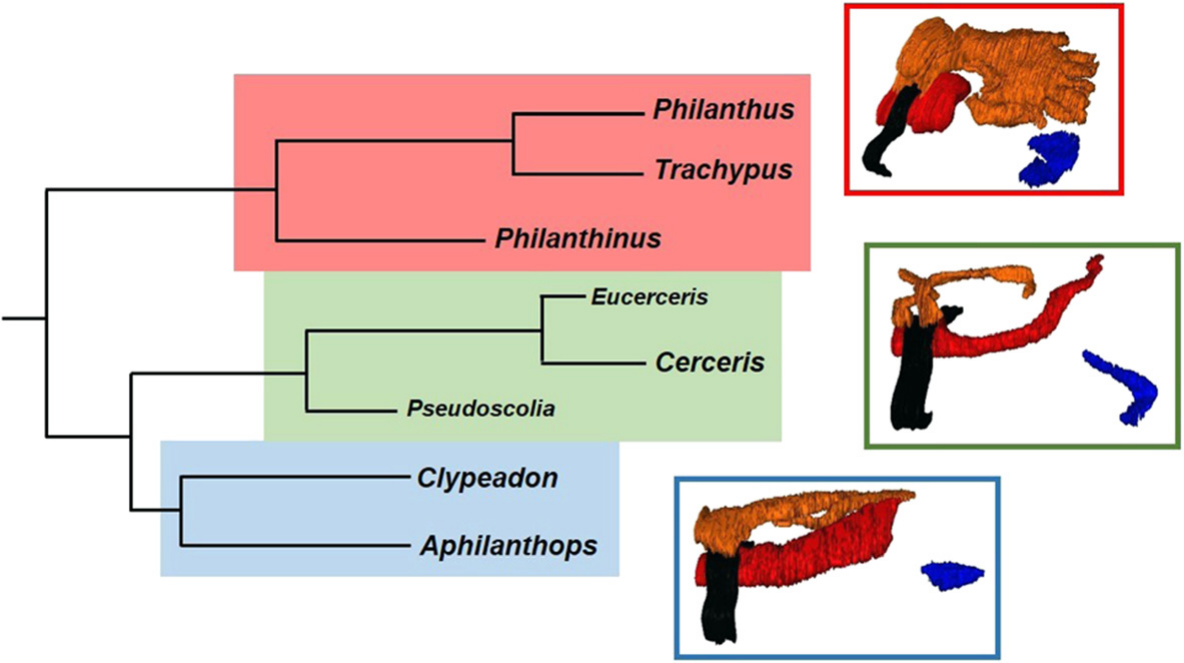
Differences in gland morphology between different tribes of the Philanthinae. The morphology of the postpharyngeal gland (PPG) differs markedly between the three tribes of the subfamily Philanthinae, with all species of the Philanthini (red) possessing complex glove-shaped glands (exemplary 3D-reconstruction: *Philanthus barbiger*), while all investigated species of both Cercerini (green; 3D-reconstruction: *Cerceris arenaria*) and Aphilanthopsini (blue; 3D-reconstruction: *Aphilanthops frigidus*) possess simple tube-shaped glands. Note that for the Cercerini the two genera *Eucerceris* and *Pseudoscolia* (small font) have not been investigated in this study. Dendrogram modified after [31]

A hierarchical cluster analysis, based on the morphological characters of the PPG, confirmed the described pattern: The first bifurcation separates the Philanthini from both Cercerini and Aphilanthopsini. Within the Philanthini, however, PPG morphology does not mirror the phylogenetic and phylogeographic relationships (e.g. North American *vs.* European/South African *Philanthus* species) within the tribe [30]. Remarkably, the shape of the PPG of female Cercerini/Aphilanthopsini resembles the PPG of the cockroach wasp *A. compressa*, a more basal taxon of the Apoidea [49], and the only other apoid wasp for which a PPG has been described [46]. Both sexes of *A. compressa* possess a simple PPG that consists of only one pair of small tubular evaginations of the pharynx and contains mostly HCs [46]. The ancestral state reconstruction of PPG shape using *A. compressa* as outgroup defined the simpler tube-shaped PPGs as the ancestral gland ‘type’ within the Philanthinae. We therefore conclude that the large complex PPGs evolved after the branching off of the Philanthini, thus representing an autapomorphy of this tribe (Fig. 9).

### Evolution of prey embalming and complex PPGs

It is tempting to speculate that, corresponding to the observed pattern for PPG size and morphology, the antimicrobial defense mechanism, involving the embalming of the prey with PPG secretion, as described in *P. triangulum* [18, 19], *P. gibbosus* (this study), and *T. elongatus* [29], has likewise evolved only in the Philanthini. The fact that both Cercerini and Aphilanthopsini possess only simple and comparatively smaller PPGs suggests that these species do not utilize the same prey preservation strategy as the Philanthini. Consistent with this view, female *A. compressa* do not use their PPGs for embalming their cockroach prey that serves as larval food. The comparatively small PPG of *A. compressa* has been proposed to rather function as a HC storage organ for other purposes [46].

There may be two possible explanations for this presumptive change in function of the PPG among basal and derived tribes of the Philanthinae. First, the Cercerini and Aphilanthopsini may use one of their two other head glands, the hypopharyngeal gland (which was present in all investigated species) or the MG, for prey embalming. The former, however, is an unlikely candidate as it lacks a reservoir (Weiss, unpublished data) and because in Hymenoptera its function seems to be restricted to the context of nutrition and digestion [50–53]. The MG of female Philanthinae, whose function is as yet unknown, does comprise a reservoir ([34, 54], this study) and was therefore included in our study. However, our results show that the MGs of female Cercerini and Aphilanthopsini are not larger than in the Philanthini, thus providing no evidence for an enlargement of the MG of the former two tribes to compensate for the smaller volume of their PPGs to allow for prey embalming. Still, as the MG does contain antimicrobial compounds in other Hymenoptera (e.g. [55–57]), a role of this gland in the antimicrobial brood defense of some Philanthinae cannot be ruled out.

Second, the development of a large gland, the production of copious amounts of HCs, and the time consuming embalming of the prey are likely to entail costs [21]. Therefore, prey embalming will only evolve under a selection regime under which its benefits outweigh these costs. The crucial factor in this regard may be the risk of fungal infestations of the larval provisions, which may depend on the microclimate in the nest as well as the susceptibility of the prey to opportunistic mold fungi.

The nest microclimate appears to be rather similar among the Philanthinae (nests in sunny sandy soil), but there are considerable differences in the prey spectrum among the Philanthinae. Members of the Philanthini provision predominantly bees and occasionally other, mostly aculeate Hymenoptera (e.g. [34]). Aphilanthopsini hunt exclusively on ants [32, 58]. Whereas some *Cerceris* species prey upon bees, most Cercerini (including the two investigated in this study) rely on Coleoptera (mainly weevils and buprestids, e.g. [33]) as larval provisions. Due to the huge differences in their ecology, these diverse prey taxa may vary in their susceptibility to microbial infestation.

Ants, the prey of the Aphilanthopsini, possess an array of elaborate individual and social immune defenses involving glandular secretions [55, 57] and hygienic behaviors (reviewed in [59]) that might render them less susceptible to both, entomopathogenic as well as opportunistic fungi. Weevils, the prey of most Cercerini, generally feed on plants that contain secondary compounds like e.g. isoflavones, terpenes, and terpenoids [60–66]. The sequestration of these compounds, which often show antimicrobial activity [64, 66], might provide a certain kind of resistance to microbial attacks.

Larval provisions of the Philanthini, by comparison, might be particularly prone to microbial attack since bees are likely to obtain a range of microorganisms while foraging on flowers e.g. via contaminated pollen [67, 68] or by transmission from other flower visitors [69, 70]. These microorganisms include specific or opportunistic pathogens of Hymenoptera [68, 70, 71] and, probably of more importance concerning the infestation risk of larval provisions, Ochratoxin and Aflatoxin producing mold fungi [67, 68]. The view that the Philanthini experience exceptional threats by microbes is supported by the fact that another antimicrobial strategy, the symbiosis with antibiotics producing *Streptomyces* bacteria [11, 72, 73], is also restricted to the tribe Philanthini [30, 74–76].

Assuming that bees are a ‘riskier’ food resource than beetles and ants, it would be compelling to further investigate the Aphilanthopsini and in particular the genus *Cerceris* for the morphology of the PPG and the implementation of prey embalming. One might expect bee-hunting *Cerceris* to exhibit some kind of prey preservation behavior similar to the Philanthini, and thus also to possess larger and more complex PPGs than their beetle-hunting congeners.

## Conclusions

All investigated species of the tribe Philanthini possess complex and large PPGs, whereas the investigated Aphilanthopsini and Cercerini bear comparably simple and smaller PPGs. Based on our findings it seems likely that all Philanthini employ prey embalming in a similar way as described for *P. triangulum* [10, 18, 19], *P. gibbosus* (this study), and *T. elongatus* [29]. In *P. triangulum*, prey embalming has significant positive effects on the females’ reproductive success by enhancing offspring survival [21]. However, the maintenance of a complex PPG and the production of high amounts of HCs for prey embalming is likely to entail costs for the female [21]. The tribe Philanthini seems to have experienced stronger selection for effective prey preservation than most species of the Aphilanthopsini and Cercerini, owing to the presumably higher susceptibility to fungal infestations of the prey taxa used as larval food, so that the fitness gains eventually outweighed the costs involved in prey embalming. Future studies on the PPG and prey preservation in the Philanthinae will shed further light on the evolution of this gland and the intriguing parental care behavior of prey embalming. Our results show that the subfamily Philanthinae provides an excellent example of how even minor differences in ecology may influence the evolution of hygienic behaviors and the related morphological characters. Given the huge diversity of mass-provisioning wasps (pompilids, ampulicids, sphecids, crabronids, eumenids [7, 33]) and their respective nesting sites and prey taxa, there are probably many more elaborate strategies of larval food preservation to be discovered.

## Additional file

Additional file 1:
**Additional methods, additional table S1 listing the bee species included in the chemical analyses, additional table S2 showing the data matrix used for the cluster analysis, additional table S3 depicting the chemical composition of the cuticular extracts of the bees and wasps, and additional figure S1 showing dendrogram of cluster analysis based on postpharyngeal and mandibular gland morphology.** (PDF 600 kb)
