# Supplementary material for: Comparative morphology of the postpharyngeal gland in the Philanthinae (Hymenoptera, Crabronidae) and the evolution of an antimicrobial brood protection mechanism
Source: BMC Evol Biol. 2015 Dec 21;15:291. doi: 10.1186/s12862-015-0565-0 (PMC4687156; doi:10.1186/s12862-015-0565-0)
Supplement: Additional file 1: — Additional methods, additional table S1 listing the bee species included in the chemical analyses, additional table S2 showing the data matrix used for the cluster analysis, additional table S3 depicting the chemical composition of the cuticular extracts of the bees and wasps, and additional figure S1 showing dendrogram of cluster analysis based on postpharyngeal and mandibular gland morphology. (PDF 600 kb) [file 12862_2015_565_MOESM1_ESM.pdf]

Additional Material:

Comparative morphology of the postpharyngeal gland in the Philanthinae (Hymenoptera, Crabronidae) and the evolution of an antimicrobial brood protection mechanism

Katharina Weiss[1], Erhard Strohm[1], Martin Kaltenpoth[2,3], Gudrun Herzner[1,*]

1 Evolutionary Ecology Group, Institute of Zoology, University of Regensburg, Universitätsstr. 31, 93053 Regensburg, Germany

2 Insect Symbiosis Research Group, Max Planck Institute for Chemical Ecology, Hans-Knoell-Str. 8, 07745 Jena, Germany

3 Present address: Johannes Gutenberg University Mainz, Institute for Zoology, Department for Evolutionary Ecology, Johann-Joachim-Becher-Weg 13, 55128 Mainz, Germany

* Corresponding author: Gudrun Herzner
Universitätsstr. 31
93053 Regensburg
Germany
Phone: +49-941-943-2997
Fax: +49-941-943-3304
Email: Gudrun.Herzner@biologie.uni-regensburg.de

Additional Methods

1. Prey embalming in *Philanthus gibbosus*

1.1 Specimens and rearing conditions

Female *P. gibbosus* were collected on flowers in Madison (Wisconsin, USA) on August 15th and 16th, 2009, and transported alive to the University of Regensburg (Bavaria, Germany). The beewolves were kept in observation cages as described earlier for *P. triangulum* [1], but because of the smaller size of *P. gibbosus*, the thickness of the sand layer in the nesting compartment was reduced to about 6 mm. The female *P. gibbosus* were supplied *ad libitum* with honey and halictid bees (Hymenoptera, Halictidae) that were collected daily in the Botanical Garden of the University of Regensburg. In order to assess whether *P. gibbosus* females embalm their prey with hydrocarbons (HCs) from their postpharyngeal gland (PPG), as has previously been described for *P. triangulum* [2,3] and *T. elongatus* [4], six paralyzed bees were removed from two artificial *P. gibbosus* brood cells (three bees each; hereafter referred to as 'provisioned bees') for chemical analysis. For comparison, nine halictid bees were collected in the field and analyzed without prior contact to *P. gibbosus* (hereafter referred to as 'control bees'). Additionally, heads of four *P. gibbosus* females that had been collected in Salt Lake City and kindly provided by Jon Seger (University of Utah) were extracted for analysis of their HC composition. The analysis of whole heads yields similar results as the analysis of dissected PPG reservoirs since the PPG contains comparatively huge amounts of HCs and their composition is virtually identical to that of the cuticle [4,5].

1.2 Molecular identification of prey species

To reliably identify the halictid bee species, we sequenced a portion of the nuclear long-wavelength rhodopsin (*lwrh*) and the mitochondrial cytochrome oxidase I (*coxI*) gene, respectively (Table S1). Briefly, after hexane extraction of samples for chemical analysis, DNA was extracted from the thorax using the Epicentre MasterPure™ DNA extraction kit (Epicentre Technologies, Madison, USA) according to the manufacturer's instructions. The DNA pellet was resuspended in 50 µl low-TE buffer (1 mM Tris/HCl, 0.01 mM EDTA). PCRs were performed on a VWR thermocycler in total reaction volumes of 12.5 µl containing 1 µl of template, 1x PCR buffer (10 mM Tris-HCl, 50 mM KCl, 0.1% Triton X-100), 2.5 mM $MgCl_2$, 240 µM deoxynucleoside triphosphates, 20 pmol of each primer, and 1 U of *Taq* DNA polymerase (VWR). The primer pairs LWRHfor1 (5'-AATTGCTATTAYGARACNTGGGT-3') / LWRHrev1 (5'-ATATGGAGTCCANGCCATRAACCA-3') and Jerry (5'-CAACATTTATTTTGATTTTTTGG-3') /

Pat (5'-TCCAATGCACTAATCTGCCATATTA-3') were used for amplification of *lwrh* and *coxI*, respectively. Cycle parameters were as follows: 3 min at 94°C, followed by 35-38 cycles of 94°C for 40 s, a primer-specific annealing temperature for 40 s (58.5°C for *lwrh* and 51°C for *coxI*), and 72°C for 40 s, and a final extension time of 4 min at 72°C. Unidirectional sequencing with primers LWRHfor1 and Jerry, respectively, was done on an ABI 3730xl capillary DNA sequencer (Applied Biosystems, USA) in the Department of Entomology at the Max Planck Institute for Chemical Ecology (Jena, Germany). Curated sequences were compared with the NCBI database using BLASTn [6].

1.3 Chemical analysis

Specimens were extracted for 10 minutes in approximately 1 ml of hexane. For the bee samples 2 µg of octadecane were added as an internal standard, to allow the quantification of the absolute amounts of the compounds. For each sample, the solvent was evaporated under a gentle stream of nitrogen, then 50-100 µl hexane were added, and the extract was transferred to a 200 µl GC-µ-vial (CZT, Kriftel, Germany). An aliquot of 1 µl of each sample was injected into a Agilent 6890N Series GC system coupled to a Agilent 5973 insert mass selective detector (Agilent Technologies, Böblingen, Germany). The GC was equipped with a nonpolar RH-5ms+ fused silica capillary column (30 m x 0.25 mm ID; df = 0.25 µm; Capital Analytical Ltd., Leeds, UK; temperature program: from 60°C to 300°C at 5°C/min and held for 1 min at 60°C and for 10 min at 300°C). Helium was used as the carrier gas, with a constant flow of 1 ml/min. A split/splitless injector was operated at 250°C in the splitless mode (60s). Electron impact mass spectra were recorded with an ionization voltage of 70 eV, a source temperature of 230°C, and an interface temperature of 315°C. The software MSD ChemStation for Windows was used for data acquisition. *N*-Alkanes were identified by the comparison of their retention times and mass spectra to those of synthetic reference substances. Linear retention indices (LRIs) for all other substances were calculated according to Van den Dool and Kratz [7] and alkenes were identified by their LRIs and mass spectra as described in Strohm *et al.* [5]. The structure of the unsaturated ketone nonacosen-6-one was tentatively assigned by its mass spectrum as described previously [4].

1.4 Data analysis

Besides the peaks that were identified as alkanes, alkenes, and alkadienes, we detected 29 additional substances in the extracts of halictid bees, none of which occurred in *P. gibbosus* samples (with the exception of one putative hexacosene, see Table S3). Since the aim of this investigation was to assess whether *P. gibbosus* females apply substances to the surface of their prey, no further efforts were

made to identify these substances, occurring only in bee samples. As the peaks of the different isomers of the alkenes in some cases were not completely separated in the GC profile, the peak areas of all isomers of a given chain length were generally combined for further analysis. The total amounts of substances for each individual sample of provisioned and control bees were calculated using the internal standard. Since the *P. gibbosus* samples had been analyzed without internal standard, absolute amounts were not calculated for this group. Additionally, the total peak area was standardized to 100% and the relative amounts of substances were calculated for each individual sample from all three groups. Inspection of the chromatograms revealed considerable differences between samples of the three groups in the peak areas of the three alkenes dominating the GC profile of female *P. gibbosus*, namely heptacosene, nonacosene, and hentriacontene. Therefore, the proportions of each of these substances were compared between provisioned and control bees. To assess whether provisioned bees carried larger amounts of HCs and a higher proportion of unsaturated HCs, we compared the total amounts of cuticular substances, as well as the relative amounts of unsaturated hydrocarbons between provisioned bees and control bees. All relative values were *arcsin*-transformed prior to analysis. All statistical comparisons were conducted with *t* tests (using test statistics for equal or unequal variances, respectively, depending on the results of preceding Levene's tests for homogeneity of variance), using the statistics software package PAST (Version 2.08b) [8].

2. Comparative morphology of head glands: Coding of character states

As the result of a comprehensive examination of both semithin histological sections and 3D-reconstructions of the head glands of female Philanthinae, we defined 13 morphological characters for the comparative analysis of the PPG and MG of the 26 philanthine species under study (Table 1, main text). For each character, different character states were categorized and numerically coded for the statistical analysis (see below; numbers in parentheses). Characters 1, 2, 7, 9, 11, and 13 are coded non-additive as their different states are considered equivalent; therefore, for these characters the numerical code does not imply a quantitative ranking of their states. Note that the two characters defined for the lower part of the PPG reservoir (characters 7 and 8) could only be defined if this part of the reservoir was present [i.e. character 1 in state (0)].

PPG:

1. O*verall structure of the PPG*.

(0) PPG consists of an upper reservoir originating from the dorsal side of the pharynx, and a lower evagination originating ventrally from the pharynx

(1) PPG consists of only the upper reservoir

2. *Shape of the upper PPG*.

(0) simple tube-shaped evagination

(1) comb-shaped: the complex upper PPG consists of multiple lobes originating from a common root with all branches being oriented to one side

(2) glove-shaped: the complex upper PPG consists of multiple 'fingers' originating from the common root of the upper PPG

(3) the complex upper PPG consists of multiple lobes originating from a common root but its form lies somewhere between (1) and (2)

3. *Number of lobes of the upper PPG (per side)*.

(0) 1

(1) < 10

(2) 10 - 15

(3) > 15

4. *Number of openings of the upper part of the PPG to the pharynx*.

(0) right and left part of the lateral symmetric upper PPG reservoir share one opening to the pharynx

(1) both right and left part of the upper PPG each have a separate opening to the pharynx

5. *Relative lateral extension of the upper PPG*. In order to exclude size effects, the lateral extension of the upper PPG was calculated in relation to the head capsule width as a measure of relative gland size. This was estimated as the proportion of the total number of semithin sections of the head capsule including compound eyes that showed structures belonging to the PPG.

(0) 45 % or less

(1) 46 - 50 %

(2) 51 - 55 %

(3) 56 - 60 %

(4) 61 - 65 %

(5) 66 - 70 %

(6) 71 - 75 %

(7) > 75 %

6. *Inner walls of the PPG*. The epithelial cells of the PPG reservoir can bear hairs on their inner side, reaching into the lumen of the gland. The density of these hairs can vary between the proximal part,

i.e. near the opening to the pharynx, and the distal parts of the gland, extending toward the compound eyes.

(0) no hairs

(1) few unevenly distributed hairs: few hairs near the opening to the pharynx but no hairs in the distal parts of the lobes

(2) few but evenly distributed hairs throughout the gland

(3) many unevenly distributed hairs: many hairs near the opening to the pharynx but only few hairs in the distal parts of the lobes

(4) many hairs, evenly distributed throughout the gland

7. *Shape of the lower PPG*.

(0) unpaired sac-shaped evagination of the pharynx

(1) paired tube-shaped evagination of the pharynx

8. *Number of openings of the lower part of the PPG to the pharynx*.

(0) right and left part of the lower PPG share one opening to the pharynx

(1) right and left part of the lower PPG each have a separate opening to the pharynx

MG:

9. *Structure of the MG*. Similar to the PPG, the MG can be formed by an upper and a lower reservoir, opening dorsally and ventrally to the mandible, respectively.

(0) only the lower part of the MG is developed

(1) only the upper part of the MG is developed

(2) both parts of the MG are developed

10. *MG size*. The volume of the MG depends on the filling status of the gland reservoir. Therefore, its size was assessed as the extension of the MG reservoir into the head capsule based on semithin histological sections and 3D-reconstructions, rather than by measuring its volume.

(0) < 1/4 of head capsule

(1) about 1/4 of head capsule

(2) 1/4 - 1/2 of head capsule

11. *Branching of the MG*.

(0) unbranched: the MG reservoir consists of one sac-like evagination

(1) branched: the MG reservoir consists of distinct branches

12. *Inner walls of the MG*. As with the PPG, the epithelial cells of the MG reservoir can bear hairs on their inner side, reaching into the lumen of the gland.

(0) no hairs

(1) few hairs

(2) many hairs

13. *Gland cells associated with the MG*. Typically the MG of Hymenoptera is associated with class III gland cells (classified according to Noirot and Quennedey [9]), that can be identified by the occurrence of end apparatuses and conducting canals. However, in some of the investigated species there are no class III gland cells associated with the MG; however, there are cells that may be classified as class I gland cells, but these cells could not unambiguously be identified as secretory cells with the applied light-microscopic methods. Hence, we distinguished only cases with class III gland cells and those without these.

(0) the MG is not associated with class III gland cells

(1) the MG is associated with class III gland cells

Additional References

1. Strohm E, Linsenmair KE. Leaving the cradle - how beewolves (*Philanthus triangulum* F) obtain the necessary spatial information for emergence. Zoology. 1994;98:137-146.

2. Herzner G, Strohm E. Fighting fungi with physics: Food wrapping by a solitary wasp prevents water condensation. Curr Biol. 2007;17:R46-R47.

3. Herzner G, Schmitt T, Peschke K, Hilpert A, Strohm E. Food wrapping with the postpharyngeal gland secretion by females of the European beewolf *Philanthus triangulum*. J Chem Ecol. 2007;33:849-859.

4. Herzner G, Kaltenpoth M, Poettinger T, Weiss K, Koedam D, Kroiss J, Strohm E. Morphology, chemistry and function of the postpharyngeal gland in the South American digger wasps *Trachypus boharti* and *Trachypus elongatus*. PLoS ONE. 2013;8.

5. Strohm E, Herzner G, Kaltenpoth M, Boland W, Schreier P, Geiselhardt S, Peschke K, Schmitt T. The chemistry of the postpharyngeal gland of female European beewolves. J Chem Ecol. 2008;34:575-583.

6. Altschul SF, Gish W, Miller W, Myers EW, Lipman DJ. Basic local alignment search tool. J Mol Biol. 2009;215:403-410.

7. Van den Dool H, Kratz PD. A generalization of the retention index system including linear temperature programmed gas-liquid partition chromatography. J Chromatogr. 1963;11:463-471.

8. Hammer Ø, Harper DAT, Ryan PD. PAST: Paleontological statistics software package for education and data analysis. Paleontol Electron. 2001;4.

9. Noirot C, Quennedey A. Fine-structure of insect epidermal glands. Annu Rev Entomol. 1974;19:61-80.

Additional Tables and Figures

Table S1 Bee species included in the chemical analysis.

Group	Species	Blast identity	Gene
**Provisioned bees**	*Lasioglossum villosulum*	98 %	*lwrh*
	Halictus kessleri	96 %	*lwrh*
	Halictus tumulorum	97 %	*lwrh*
	Halictus simplex	97 %	*lwrh*
	Halictus tumulorum	97 %	*lwrh*
	Halictus tumulorum	98 %	*lwrh*
**Control bees**	*Halictus pseudomaculatus*	97 %	*lwrh*
	Halictus kessleri	97 %	*lwrh*
	Halictus kessleri	96 %	*lwrh*
	Lasioglossum villosulum	99 %	*lwrh*
	not identified	---	---
	Halictus kessleri	96 %	*lwrh*
	Halictus tumulorum	99 %	*coxI*
	Halictus kessleri	97 %	*lwrh*
	Halictus kessleri	96 %	*lwrh*
	Halictus kessleri	96 %	*lwrh*
	Halictus scabiosae	97 %	*lwrh*

Species identity of halictid bees taken from *P. gibbosus* brood cells (provisioned bees) and control bees that had no contact to the beewolves (control bees) as established by comparison of gene sequences with the NCBI database using BLASTn (Blast identity). Abbreviations: *lwrh*, nuclear long-wavelength rhodopsin gene; *coxI*, mitochondrial cytochrome oxidase I gene.

Table S2 Character matrix for the statistical analysis of head gland morphology of female Philanthinae.

	PPG characters								MG characters				
**Taxon**	**1**	**2**	**3**	**4**	**5**	**6**	**7**	**8**	**9**	**10**	**11**	**12**	**13**
Aphilanthops frigidus	0	0	0	0	4	3	1	1	1	1	0	0	0
Clypeadon laticinctus	0	0	0	1	2	1	1	1	1	1	0	0	0
Cerceris arenaria	0	0	0	1	3	3	1	1	1	1	0	0	1
Cerceris quinquefasciata	0	0	0	1	0	4	1	1	1	1	0	0	1
Philanthinus quattuordecimpunctatus	0	2	1	0	4	0	0	0	1	1	0	0	0
Trachypus flavidus	1	2	2	?	6	3	---	---	?	?	?	?	1
Trachypus elongatus	0	2	2	0	6	3	0	0	1	1	0	0	0
Trachypus patagonensis	0	?	?	?	?	?	0	?	1	1	0	0	0
Trachypus boharti	1	1	2	1	7	3	---	---	1	1	0	0	1
Philanthus venustus	0	2	2	0	4	1	0	0	1	1	0	0	0
Philanthus triangulum	0	2	?	1	?	3	0	0	1	2	0	0	1
Philanthus triangulum diadema	0	2	2	1	5	3	0	0	1	1	0	0	1
Philanthus capensis	0	2	2	1	5	3	0	0	1	1	0	0	0
Philanthus loefflingi	0	2	2	1	6	1	0	0	1	1	0	0	0
Philanthus rugosus	0	2	2	1	4	3	0	0	1	2	0	0	0
Philanthus melanderi	0	3	2	1	3	3	0	0	1	2	0	0	1
Philanthus coronatus	0	2	2	1	7	3	0	0	1	0	0	0	1
Philanthus bicinctus	0	3	3	1	?	?	0	0	1	0	0	0	0
Philanthus ventilabris	0	2	2	0	4	3	0	0	1	1	0	0	0
Philanthus crabroniformis	0	?	?	?	?	?	0	?	1	1	0	0	0
Philanthus multimaculatus	0	2	2	0	?	0	0	0	1	1	0	0	0
Philanthus barbiger	0	2	2	0	5	1	0	0	1	1	0	0	0
Philanthus gibbosus	0	2	1	1	2	3	0	0	1	0	0	0	1
Philanthus albopilosus	0	3	2	1	5	3	0	0	1	0	0	0	1
Philanthus psyche	0	2	3	?	?	3	0	0	1	0	0	0	0
Philanthus pulcher	0	?	?	1	?	3	0	0	1	?	?	0	0

The numbering of the morphological characters of the PPG and the MG and the numeric coding of the character states correspond to the description in section 2 above. ?, character state could not be determined; ---, character not present in this species.

Table S3 Chemical composition of the cuticular extracts of *P. gibbosus* females, provisioned bees, and control bees.

Substance	LRI	P. gib	PB	CB	Diagnostic ions
Unidentified 01	2052	-	+	+	
**Heneicosadiene 1**	**2069**	**-**	**+**	**+**	**292**
**Heneicosadiene 2**	**2081**	**-**	**+**	**+**	**294**
***n*-Heneicosane**	**2100**	**-**	**+**	**+**	**296**
Unidentified 02	2138	-	+	+	
Unidentified 03	2148	-	-	+	
Unidentified 04	2166	-	-	+	
Unidentified 05	2175	-	-	+	
Unidentified 06	2194	-	-	+	
Unidentified 07	2201	-	-	+	
Unidentified 08	2238	-	-	+	
Unidentified 09	2248	-	+	+	
**Tricosadiene**	**2251**	**-**	**-**	**+**	**320**
Unidentified 10	2259	-	+	-	
**Tricosene 1**	**2274**	**-**	**+**	**+**	**322**
**Tricosene 2**	**2281**	**-**	**+**	**+**	**322**
Unidentified 11	2288	-	-	+	
***n*-Tricosane**	**2300**	**+**	**+**	**+**	**324**
Unidentified 12	2338	-	+	-	
Unidentified 13	2341	-	+	+	
Unidentified 14	2351	-	+	+	
**Tetracosene**	**2370**	**-**	**-**	**+**	**334**
Unidentified 15	2394	-	-	+	
***n*-Tetracosane**	**2402**	**+**	**+**	**+**	**338**
Unidentified 16	2428	-	-	+	
Unidentified 17	2433	-	+	+	
Unidentified 18	2446	-	+	+	
Unidentified 19	2460	-	+	+	
**Pentacosadiene**	**2470**	**+**	**+**	**+**	**348**
**Pentacosene 1**	**2475**	**+**	**+**	**+**	**350**
**Pentacosene 2**	**2482**	**+**	**+**	**+**	**350**
**Pentacosene 3**	**2493**	**+**	**+**	**+**	**350**
***n*-Pentacosane**	**2500**	**+**	**+**	**+**	**352**
Unidentified 20	2542	-	+	+	
Unidentified 21	2553	-	+	+	
**Hexacosene 1**	**2577**	**+**	**-**	**-**	**364**
Unidentified 22 [1]	**2584**	**+**	**+**	**-**	
Unidentified 23	2594	-	-	+	
***n*-Hexacosane**	**2602**	**-**	**+**	**+**	**366**
Unidentified 24	2662	-	+	+	
**Heptacosene 1**	**2672**	**-**	**-**	**+**	**378**
**Heptacosadiene + Heptacosene 2**	**2676**	**+**	**+**	**+**	**376, 378**

**Heptacosene 3**	**2684**	**+**	**+**	**+**	**378**
**Heptacosene 4**	**2694**	**+**	**+**	**+**	**378**
***n*-Heptacosane**	**2700**	**+**	**+**	**+**	**380**
Unidentified 25	2743	-	+	+	
Unidentified 26	2755	-	+	-	
**Octacosene**	**2777**	**+**	**+**	**-**	**392**
**Nonacosene 1**	**2859**	**-**	**+**	**+**	**406**
**Nonacosene 2**	**2865**	**-**	**+**	**+**	**406**
**Nonacosene 3**	**2878**	**+**	**+**	**+**	**406**
**Nonacosene 4**	**2886**	**+**	**+**	**+**	**406**
***n*-Nonacosane**	**2900**	**+**	**+**	**+**	**408**
**Hentriacontadiene 1**	**3019**	**-**	**-**	**+**	**432**
**Hentriacontadiene 2**	**3026**	**-**	**-**	**+**	**432**
**Hentriacontadiene 3**	**3044**	**-**	**-**	**+**	**432**
**Hentriacontadiene 4**	**3051**	**-**	**+**	**+**	**432**
**Nonacosen-6-one**	**3060**	**+**	**+**	**-**	**99, 349, 420**
**Hentriacontene 1**	**3067**	**+**	**+**	**+**	**434**
**Hentriacontene 2**	**3075**	**-**	**+**	**-**	**434**
Unidentified 27	3087	-	+	+	
Unidentified 28	3146	-	-	+	
**Tritriacontadiene 1**	**3192**	**-**	**-**	**+**	**462**
**Tritriacontadiene 2**	**3222**	**-**	**+**	**-**	**462**
**Tritriacontadiene 3**	**3231**	**-**	**-**	**+**	**462**

[1] Based on the available partial mass spectrum and the comparison of its LRI to published data, this very minor compound, that occurred only in samples of *P. gibbosus* females and provisioned bees, most likely is a hexacosene. However, it could not unambiguously be identified due to the lack of the molecular ion in the mass spectrum and consequently is classified as unidentified. It is therefore not discussed in the main text.

Abbreviations: P.gib, *P. gibbosus* female heads; PB, provisioned bees; CB, control bees; LRI, linear retention index (calculated in relation to *n*-alkanes). +, substance detected in at least one sample of the group; -, substance not detected. Substances shaded in grey only occurred in samples of *P. gibbosus* heads and on provisioned bees taken from *P. gibbosus* brood cells, but not on control bees.

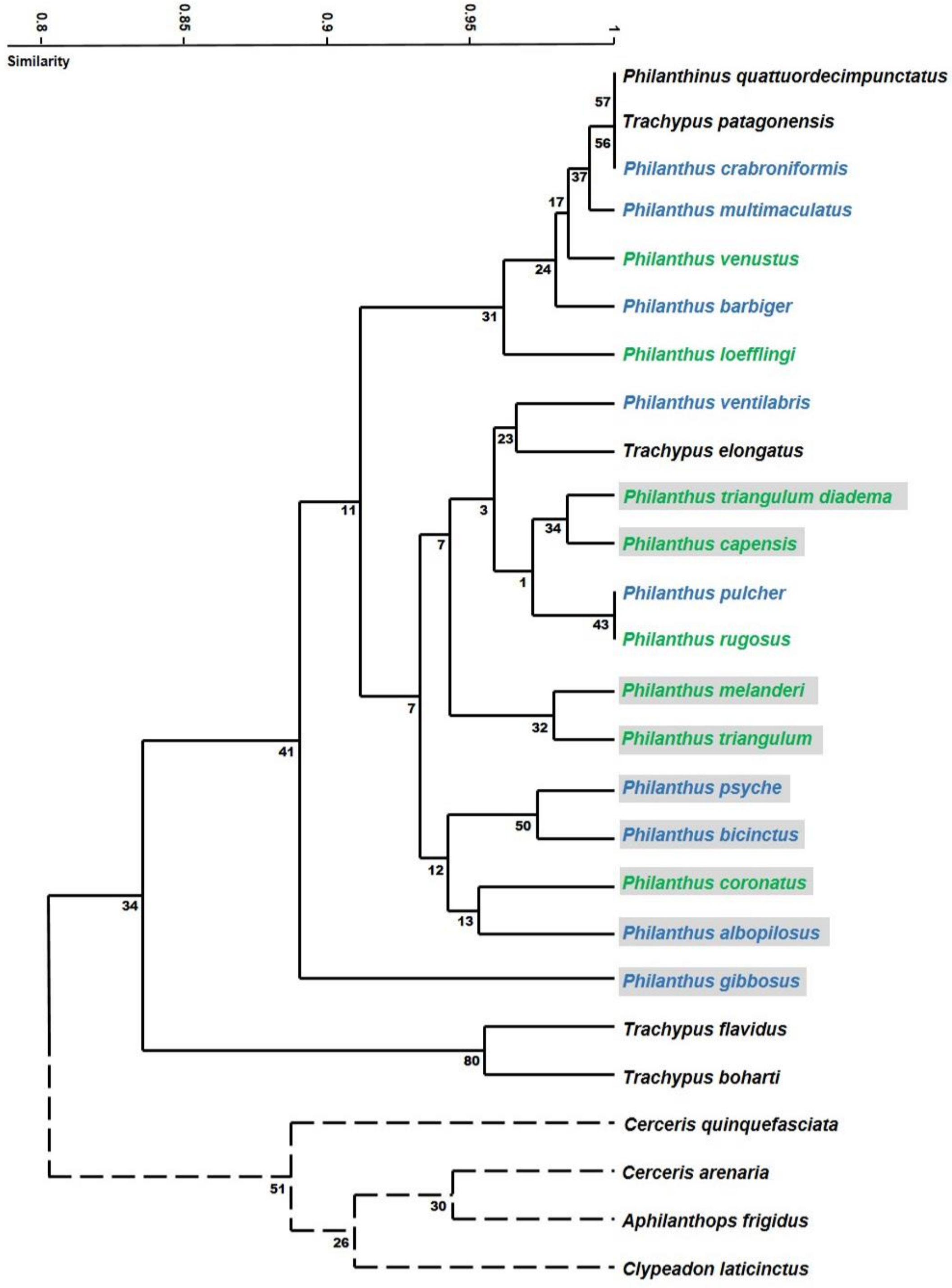

Figure S1 Dendrogram of Philanthinae based on postpharyngeal and mandibular gland morphology. Dendrogram based on the hierarchical cluster analysis of all 13 morphological characters defined for the PPG and MG of female Philanthinae. Values at the nodes are bootstrap values. Solid lines indicate members of the tribe Philanthini, dashed lines indicate members of the tribes Cercerini and Aphilanthopsini. Names of European and South African *Philanthus* species are printed in green; names of North American *Philanthus* species are printed in blue. The grouping of species shaded in gray differs from the cluster analysis of only the eight morphological characters of the PPG (Figure 7, main text). Bray-Curtis was used as a similarity measure; N bootstrap replicates = 10,000.
